# The role of cytochrome P450 enzymes in carcinogen activation and detoxication: an *in vivo–in vitro* paradox

**DOI:** 10.1093/carcin/bgy058

**Published:** 2018-05-03

**Authors:** Lindsay Reed, Volker M Arlt, David H Phillips

**Affiliations:** 1 Department of Analytical, Environmental and Forensic Sciences, MRC-PHE Centre for Environment and Health, King’s College London, Franklin-Wilkins Building, London, UK; 2 NIHR Health Protection Unit in Health Impact of Environmental Health Hazards at King’s College London in Partnership with Public Health England, London, UK

## Abstract

Many chemical carcinogens require metabolic activation via xenobiotic-metabolizing enzymes in order to exert their genotoxic effects. Evidence from numerous *in-vitro* studies, utilizing reconstituted systems, microsomal fractions and cultured cells, implicates cytochrome P450 enzymes as being the predominant enzymes responsible for the metabolic activation of many procarcinogens. With the development of targeted gene disruption methodologies, knockout mouse models have been generated that allow investigation of the *in-vivo* roles of P450 enzymes in the metabolic activation of carcinogens. This review covers studies in which five procarcinogens representing different chemical classes, benzo[*a*]pyrene, 4-aminobiphenyl (4-ABP), 2-amino-1-methyl-6-phenylimidazo[4,5-*b*]pyridine, 2-amino-9*H*-pyrido[2,3-*b*]indole and 4-(methylnitrosamino)-1-(3-pyridyl)-1-butanone, have been administered to different P450 knockout mouse models. Paradoxically, while *in-vitro* studies using subcellular fractions enriched with P450 enzymes and their cofactors have been widely used to determine the pathways of activation of carcinogens, there is evidence from the *in-vivo* studies of cases where these same enzyme systems appear to have a more predominant role in carcinogen detoxication rather than activation.

## Teaser

Cytochrome P450 enzyme systems have been widely used *in vitro* to determine the pathways of activation of procarcinogens, but paradoxically, these same enzymes can play a more predominant role in carcinogen detoxification *in vivo*.

## Introduction

Mammalian cytochrome P450 (CYP) enzymes are a superfamily of haemoproteins. They have many roles including cholesterol metabolism and steroidogenesis; however, one particularly important role is in the metabolism of xenobiotics, with P450 enzymes accounting for 70–80% of oxidation metabolizing enzymes (1). They make up part of the mixed-function oxidase system along with other enzymes including NADPH:cytochrome P450 reductase (POR), NADH:cytochrome *b*_*5*_ reductase (Cyb5R) and the haemoprotein cytochrome *b*_*5*_ (Cyb5) (2,3). Although the majority of P450 reactions involve the introduction of polar groups to parent compounds to enable detoxication and excretion from the body, they have also been implicated in the bioactivation of carcinogens. Many carcinogens are considered procarcinogens that require metabolic activation to exert their genotoxic effects (4). Oxidative activation of carcinogens by P450 enzymes leads to the formation of electrophilic reactive intermediates that can bind to DNA, giving rise to DNA adducts that potentially cause mutations (5). *In-vitro* systems have played a central role in investigating carcinogen activation and have included microsomal fractions, cell culture and reconstituted systems (6–8). More recently, the development of targeted gene disruption methodologies (9,10) has given rise to mice that do not express a particular CYP isoenzyme (11–13). Studies with these mice can provide mechanistic insights into the contribution of P450 enzymes to the activation and detoxication *in vivo* of xenobiotics in general and of carcinogens in particular (14). However, findings from such *in-vivo* studies of carcinogen activation are at odds with what might be expected from the evidence obtained from *in-vitro* studies. In what follows, we describe these paradoxical findings for five well-known and widely studied carcinogens, representing different chemical classes.

## Benzo[*a*]pyrene

**Figure 1.**
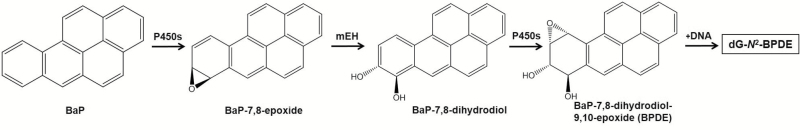
P450-mediated bioactivation pathway of BaP.

On the basis of numerous *in vitro* studies, benzo[*a*]pyrene (BaP) is considered a procarcinogen activated via P450-dependent monooxygenases, with CYP1A1 and CYP1B1 playing major roles in the bioactivation pathway (15–17). The two enzymes catalyse the initial oxidation of BaP to form BaP-7,8-epoxide, which is then converted to BaP-7,8-dihydrodiol by epoxide hydrolase. BaP-7,8-dihydrodiol then undergoes further bioactivation by CYP1A1 and CYP1B1 to form the ultimately reactive species, BaP-7,8-dihydrodiol-9,10-epoxide (BPDE) (18,19) (Figure 1). BPDE is able to react with DNA, preferentially at guanine residues, to form primarily the premutagenic adduct 10-(deoxyguanosin-*N*^2^-yl)-7,8,9-trihydroxy-7,8,9,10-tetrahydro-BaP (dG-*N*^2^-BPDE) (20–23). The necessity for P450 activity for BaP activation *in vitro* has been confirmed in many studies where P450 activity in cells correlates with cytotoxicity, where inhibition of P450s reduces toxicity and in test systems where metabolism, macromolecular binding and mutagenicity are all dependent on the presence of P450 activity, e.g. in the Ames Salmonella mutation assay (24–27).

Indications of the complexity of BaP metabolism in *in-vivo* studies have emerged with the use of P450 knockout models. For example, using the Cre-*lox* system, *Cyp1a1(−/−*) knockout mice on a C57BL/6J and 129/J background were developed through the deletion of the *Cyp1a1* gene (28). These mice are described as being viable and show no obvious phenotype compared with wild-type littermates. In an initial study, *Cyp1a1(+/−*) and *Cyp1a1(−/−*) mice were treated with a single i.p. dose of 500 mg/kg body weight (bw) BaP in order to explore the role of CYP1A1 in BaP-mediated toxicity (29). It was hypothesized that *Cyp1a1(−/−*) mice would have greater protection than their *Cyp1a1(+/−*) heterozygous littermates against liver damage and hepatic BaP–DNA adduct formation. However, the formation of hepatic BaP–DNA adducts in *Cyp1a1(−/−*) mice was 4-fold higher compared with the *Cyp1a1(+/−*) mice. In order to assess whether other inducible enzymes were able to contribute to BaP activation, mice were pretreated with tetrachlorodibenzo-*p*-dioxin (TCDD). These TCDD-pretreated mice were shown to have decreased levels of hepatic BaP–DNA adducts and enhanced clearance of BaP from the blood, indicating that the accumulation of BaP–DNA adducts in *Cyp1a1(−/−*) mice could be due to the lack of Cyp1a1-mediated detoxication (29). One suggested explanation for the results was the potential involvement of cyclooxygenase-2 (PTGS2), a BaP-inducible enzyme, that has been implicated in the activation of BaP to reactive intermediates (30,31). Another suggestion was that CYP1A1 is actually more important for the detoxication of BaP, metabolizing BaP to BaP phenols, quinones and oxides that can be conjugated enzymatically to form readily excretable products (32). *Cyp1a1(+/−*) mice in this study were shown to be protected against BaP–DNA adduct formation by the induction of CYP1A1 and the more rapid clearance of BaP (29). In order to investigate this paradoxical result, further studies were carried out, this time using *Cyp1a1(+/+*) wild-type mice as a comparison instead of the heterozygous *Cyp1a1(+/−*) mouse (33). DNA adduct formation correlated with the results of the previous study as the BaP-treated *Cyp1a1(−/−*) mice formed significantly higher levels of BaP–DNA adducts in the livers, spleen and marrow compared with wild-type mice when treated with three oral doses of 12.5 or 125 mg/kg bw for 18 days. Pharmacokinetic studies showed that the clearance rate of BaP was four times slower in *Cyp1a1(−/−*) mice compared with wild-type mice. Pretreatment with TCDD before BaP administration decreased the half-life of BaP by half in wild-type mice, whereas the half-life in the TCDD-pretreated *Cyp1a1(−/−*) mice was unaffected, demonstrating that the clearance of BaP appears to be dependent on Cyp1a1. A comparison of survival rates between *Cyp1a1(−/−*) and wild-type mice gave further evidence of the protective role of Cyp1a1; an oral dose of 125 mg/kg bw BaP was lethal to *Cyp1a1(−/−*) mice by 30 days (33).

The mechanism for this enhanced sensitivity was further investigated with *Cyp1a1(−/−), Cyp1a2(−/−*) and *Cyp1b1(−/−*) single knockout mice and with *Cyp1a1/1b1(−/−*) and *Cyp1a2/1b1(−/−*) double knockout mice compared with *Cyp1a(+/+*) mice (34). Oral treatment with 12.5 mg/kg bw BaP resulted in significantly higher BaP–DNA adduct formation in the liver, spleen and bone marrow of *Cyp1a1(−/−*) mice and 125 mg/kg bw BaP resulted in significantly higher BaP–DNA adduct formation in the small intestine, spleen and bone marrow. *Cyp1a1/1b1(−/−*) mice had fewer adducts in the small intestine for both doses of BaP compared with wild-type, and although BaP–DNA adduct formation was higher in the liver, spleen and bone marrow at the lower dose, administration of the higher dose resulted in fewer DNA adducts in these organs compared with wild type. The pharmacokinetic studies were repeated as before and, regardless of TCDD pretreatment, Cyp1a1 was the primary determinant of BaP clearance. Cyp1b1, however, appeared to be responsible for metabolic activation of BaP in the spleen and bone marrow, resulting in immune damage in the absence of Cyp1a1. The presence of significantly higher levels of BaP–DNA adduct formation in the *Cyp1a1/1b1(−/−*) mice compared with wild-type mice could mean either that other P450 isoenzymes are involved in BaP activation or that there is a P450-independent activation mechanism for BaP (34,35).

Because the cytochrome P450 family is large, with many of its members having overlapping substrate specificity, determining the *in-vivo* role of individual P450 enzymes is difficult (36). In order for P450 enzymes to catalyse reactions, they must receive electrons from electron donors, the predominant one being POR (4). Systemic knockout of POR in mice utilizing the Cre/loxP system results in embryonic lethality due to the requirement for P450 expression during development (37). However, hepatic cytochrome P450 reductase null (HRN) mice, in which POR is deleted specifically in hepatocytes, are viable and exhibit no overt phenotypical differences from wild-type mice other than having steatotic livers as a consequence of non-functioning P450-housekeeping activity involved in cholesterol metabolism (12). In a study investigating BaP activation, HRN mice were compared with wild-type mice homologous for the loxP sites at the *Por* locus (*Por*^*lox/lox*^) (20). Microsomal fractions isolated from the livers of BaP-treated and -untreated HRN and wild-type mice were used to assess BaP activation in *in-vitro* incubations with calf thymus DNA and the enzymatic cofactor NADPH. DNA adduct formation was ∼4-fold higher in incubations with hepatic microsomal fractions from BaP-pretreated wild-type mice and 7-fold higher in the microsomal fractions from untreated wild-type mice relative to those from HRN mice. Microsomal incubations were also carried out in the presence of inhibitors of POR (α-lipoic acid), Cyp1a1/1a2 (α-naphthoflavone) and Cyp1a1 (ellipticine) and showed that DNA binding was reduced overall by 70–90%, suggesting that activation of BaP *in vitro* is mostly attributable to Cyp1a enzyme activity in both mouse strains. However, this did not correlate with the *in-vivo* findings, where exposure to a single i.p. dose of 125 mg/kg bw BaP resulted in ~13-fold ‘higher’ level of BaP–DNA adducts in the livers of HRN mice relative to wild-type mice, as well as significantly higher levels of DNA adducts in extra-hepatic tissues such as lung, forestomach, glandular stomach, kidney, spleen and colon. These findings mirror those of the *Cyp*-knockout mice studies mentioned above (29,33,34) except that here no statistically significant difference in the clearance of BaP from blood was found between HRN and wild-type mice (20).

During these studies, it was observed that HRN mice expressed significantly higher levels of the protein Cyb5, another electron donor to P450 enzymes. The hepatic cytochrome b5/P450 reductase null (HBRN) mouse, which lacks both hepatic Cyb5 and POR, was used alongside the HRN mouse to assess whether the induction of Cyb5 was responsible for the increased levels of adducts in the HRN mice (38). Microsomal fractions isolated from wild-type, HRN and HBRN mice showed that the activation of BaP *in vitro* was reduced as electron donors were lost, suggesting a role for Cyb5 in the activation of BaP *in vitro*. *In-vivo* data, however, showed that levels of hepatic BaP–DNA adduct formation were again higher (~7-fold) in HRN mice, and while levels in HBRN mice were significantly lower than in HRN mice, they were not significantly different to those in wild-type mice.

In order to investigate the role of extra-hepatic organs and alternative routes of administration, another study was carried out to study the effects of the first-pass elimination, utilizing both oral and i.p. administration of BaP (39). BaP–DNA adduct formation in HRN mice was dose-dependent by both routes. Intraperitoneal administration at 12.5 and 125 mg/kg bw resulted in a 8- to 10-fold increase in DNA adduct formation in the livers of HRN mice relative to wild-type, correlating with the previous study (20). DNA adduct formation after oral treatment was lower than by i.p. treatment at both doses, but hepatic BaP–DNA adduct formation was still higher (~2-fold) in HRN mice than in wild-type. The fold increase in both cases was lower overall than with i.p. administration, indicating that after oral administration, the first-pass metabolism of BaP occurs in the gastrointestinal tract. As POR is deleted only in the hepatocytes of HRN mice, immunohistochemical staining was used to determine whether non-hepatocytes were contributing to the elevated levels of DNA adducts (i.e. dG-*N*^2^-BPDE) in liver; no differences in BaP–DNA adduct formation were observed between POR-deficient hepatocytes and POR-proficient non-hepatocytes, showing that hepatocytes possessed ample capacity for the formation of BaP-derived DNA adducts (39).

The reductase conditional null (RCN) mouse is a variant on the HRN mouse line *(Por*^*lox/lox*^*/Cre*^*CYP1A1*^), whereby POR can be deleted conditionally in the liver and gastrointestinal tract using the rat CYP1A1 promoter to drive Cre recombinase expression (13). Administration of the CYP1A1 inducers TCDD or β-naphthoflavone results in deletion of both hepatic and gastrointestinal POR, whereas administration of 3-methylcholanthrene (3MC) results in the loss only of hepatic POR expression (13,40). RCN mice were treated with 40 mg/kg bw 3MC i.p. 2 weeks prior to BaP treatment with 125 mg/kg bw, with control mice receiving no 3MC (39). The conditional nature of the RCN mouse means it is able to act as its own control. There was, again, higher BaP–DNA adduct formation (~6-fold) in the livers of RCN + 3MC mice than in controls (RCN–3MC mice). Analysis also showed significantly higher levels of BaP–DNA adduct formation (~2-fold) in the lungs, glandular stomach, kidney, spleen and colon of RCN + 3MC mice than of RCN–3MC mice. Investigations were then carried out *in vitro* to elucidate the participation of the electron donors in the activation pathway. Although POR is the predominant electron donor to P450 enzymes, Cyb5 can also act as an electron donor to P450 enzymes *in vivo* in conjunction with the enzyme Cyb5R (41). It was observed that Cyb5 protein expression was marginally, but detectably, higher (∼1.3-fold) in the hepatic microsomal fractions of HRN mice compared with wild-type after BaP treatment once daily for 5 days (39). Thus, the increased BaP–DNA adduct formation in the livers of HRN mice could arise from Cyb5 compensating for the loss of POR and maintaining a certain level of hepatic P450 activity. *In-vitro* studies using reconstituted systems containing CYP1A1 and different ratios of POR and Cyb5 have shown that Cyb5 can stimulate CYP1A1-mediated BaP–DNA adduct formation, indicating that Cyb5 can participate in the electron transfer from NADPH to CYP1A1 required for enzyme activity (42) and that the NADH/Cyb5R/Cyb5 system can act as sole electron donor to catalyse CYP1A1-mediated BaP bioactivation (43). All these findings indicate that even low POR expression in the livers of HRN mice with the induction of Cyp1a1 and Cyb5 by BaP might be sufficient for efficient BaP bioactivation *in vivo* (42). They also suggest that NADH-dependent Cyb5R can replace NADPH-dependent POR in the CYP1A1-catalysed activation of BaP (43), which correlates with the observation that hepatic microsomal incubations carried out in the presence of NADH instead of NADPH still resulted in BaP–DNA adduct formation (20). Whether or not this is sufficient to explain the formation of higher levels of BaP–DNA adducts in HRN and RCN mice than in wild-type mice is not clear. One other study utilized a variant of the HRN mouse, the HRN-*gpt* mouse (44–46), a cross between HRN and *gpt* delta mice that enabled the use of a gene mutation assay in the investigation of hepatic P450-catalysed bioactivation in order to assess the mutagenicity of BaP in the absence of hepatic P450 activity (47). HRN-*gpt* and wild-type mice were treated i.p. with 50 mg/kg bw BaP once a day for four consecutive days and sacrificed 2 weeks after the last treatment. The *gpt* gene mutation assay showed that BaP induced a ‘higher’ mutation frequency in both the liver and extra-hepatic tissues of HRN-*gpt* mice compared with wild-type mice. In order to investigate these results, further S9 fractions were isolated and used to screen activity of other potential enzymes. Although the results suggested that P450s were the predominant activating enzymes present, inhibitors of aldo-keto reductase, COX1/2 and 5-LOX all significantly reduced the level of BPDE–DNA adduct formation using HRN-*gpt* S9 *in vitro*, further supporting evidence for P450-independent BaP activation (47). Although the use of *in vitro* systems provides mechanistic insights, full extrapolation to microsomes or even *in-vivo* situations may not be straightforward.

The aryl hydrocarbon receptor (AHR) is important in the metabolic activation of BaP as the binding of BaP to the receptor results in an induction of metabolizing enzymes, including CYP1A1 and 1B1. This led to an investigation in *Ahr*-knockout mice, developed using homologous recombination in embryonic stem cells (48) with the hypothesis that *Ahr(−/−*) mice would be less susceptible to the genotoxic effects of BaP (49). Exposure to 100 µmol/kg bw BaP i.p. induced Cyp1a1/1a2 in hepatic microsomes isolated from wild-type mice with an increase in both protein expression and enzyme activity but not in those isolated from *Ahr(−/−*) mice, where enzyme activity was markedly lower due to Ahr-dependent mechanisms of Cyp1a1/1a2 induction. Despite the absence of the Ahr, a number of hepatic BaP-derived DNA adducts were formed in the *Ahr(−/−*) mice. There are other P450 enzymes, e.g. CYP2C, and non-P450 enzymes, e.g. PTGS-2, which are regulated by Ahr-independent mechanisms that could be contributing to the activation of BaP (49). In order to investigate these findings further, an *Ahr*(+/*−*) mouse model was employed alongside the *Ahr(+/+*)and *Ahr(−/−*) mice used previously (50). After exposure to 100 mg/kg bw BaP by oral gavage, DNA adducts, protein adducts, metabolites, conjugates and unmetabolized BaP were measured, and all were found to be at higher levels in the *Ahr(−/−*) mice than in both the *Ahr(+/−*) and *Ahr(+/+*) mice. The levels of unmetabolized BaP were highest in the distal organs like the lungs and spleen and lowest in the liver. The lower metabolic clearance of BaP in the *Ahr(−/−*) mice could be attributed to reduced metabolism in the liver as Ahr-dependent Cyp1a1/1a2 induction is absent, and therefore, presystemic elimination through the gut is reduced. BaP metabolism could be due to the constitutive expression of Cyp1b1 or to an Ahr-independent mechanism (50). To address this issue further, a time-course experiment was carried out with *Ahr(+/+*) and *Ahr(−/−*) mice (51). The mice were exposed to a single oral dose of 100 mg/kg bw BaP, after which *Ahr(−/−*) mice exhibited higher levels of pulmonary BaP–DNA adducts and protein adducts in the liver, lung, spleen, heart and kidney over time; however, the rate of biotransformation was again slower with higher levels of unmetabolized BaP in all major tissues correlating with the previous time-course experiment (50). Collectively, these studies demonstrate that the lack of functional Ahr results in slower clearance of BaP (50,51), which correlates with the findings with *Cyp1a1(−/−*) mice (33), but not those with HRN mice (20).

Thus, the increases in hepatic BaP–DNA adduct formation in *Cyp1a1(−/−*), HRN, RCN and *Ahr(−/−*) mice (20,29,33,34,39,49–51) all provide an anomaly: *in-vitro* use of hepatic enzyme systems show that BaP is metabolically ‘activated’ by cytochrome P450s, and yet *in vivo* hepatic P450 enzymes appear to play a more pivotal role in BaP detoxication rather than its activation, with the potential for a P450/Ahr-independent mechanism for the activation of BaP.

## 4-Aminobiphenyl

**Figure 2.**
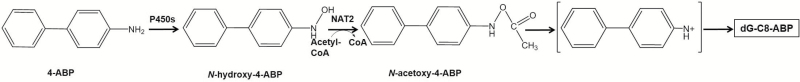
P450-mediated bioactivation pathway of 4-ABP.

4-Aminobiphenyl (4-ABP) is an environmental carcinogen that is metabolized primarily in the liver to *N*-hydroxy-4-ABP, the precursor to 4-ABP-derived DNA adduct formation (52) (Figure 2). The primary step in the activation pathway is *N*-hydroxylation catalysed by CYP1A2, as has been demonstrated *in vitro* by numerous methods including microsomes and purified P450 enzymes from human and rat livers (52–57). Metabolic activation of *N*-hydroxy-4-ABP is by esterification mediated by conjugation enzymes such sulfotransferases or *N*-acetyltransferases (53,58). An *in-vitro* study utilizing Hepa1c1c7 cells transfected with mouse *Cyp1a2* and *Cyp2e1* expression plasmids demonstrated the ability of recombinant mouse Cyp1a2 and Cyp2e1 to *N*-hydroxylate 4-ABP in living cells to produce increased levels of *N*-hydroxy-4-ABP compared with wild-type cells (59). Enzyme kinetic studies carried out with liver microsomes from *Cyp1a2(−/−*) and *Cyp2e1(−/−*) mice showed a significant contribution from Cyp2e1 toward 4-ABP *N*-hydroxylation in male mouse microsomes and a contribution from both Cyp2e1 and Cyp1a2 in female mouse microsomes (59).

Investigation of 4-ABP metabolism *in vivo* in both *Cyp1a2(−/−*) and *Ahr(−/−*) mice showed that treatment of mice with 600 or 1200 nmol in two doses at 8 and 15 days of age 4-ABP induced hepatocellular adenoma and liver foci; however, there were no differences found in incidence between *Cyp1a2(−/−), Ahr(−/−*) and wild-type mice, irrespective of the dose (60). Hepatic microsomal fractions isolated from *Cyp1a2(−/−*) and *Ahr(−/−*) mice exhibited half the microsomal arylamine *N*-hydroxylation activity of that seen with wild-type hepatic microsomal fractions during incubations with 4-ABP, indicating that only half of the enzymatic activity attributed to 4-ABP activation was due to Cyp1a2 (60).

Another study using *Cyp1a2(−/−*) mice involved topical applications of 100 µmol/kg bw 4-ABP, with or without TCDD pretreatment, and measurement of methaemoglobin levels as a biomarker of 4-ABP exposure (61). It was expected that 4-ABP-induced methaemoglobin formation would be higher in mice pretreated with TCDD due to the induction of Cyp1a2. It was found, however, that the presence of Cyp1a2 actually decreased methaemoglobin formation, and in fact, TCDD pretreatment lowered it further (61).

A third *in-vivo* study using *Cyp1a2(−/−*) mice was carried out with topical applications of 10 mg/kg bw 4-ABP with or without TCDD pretreatment, this time focusing on DNA adduct formation (62). The same expectation was held, that mice possessing Cyp1a2, particularly those having been pretreated with TCDD, would form higher levels of 4-ABP-DNA adducts than mice lacking Cyp1a2. However, the opposite effect was again observed. *Cyp1a2(−/−*) mice formed similar or higher levels of 4-APB-DNA adducts than wild-type mice in both liver and bladder. When mice were pretreated with TCDD, hepatic DNA adduct levels were either lower or similar to the corresponding group of mice that did not receive TCDD, adding further weight to the argument that Cyp1a2 is more important for clearance of 4-ABP than for its activation (62).

## 2-Amino-1-methyl-6-phenylimidazo[4,5-*b*]pyridine

**Figure 3.**
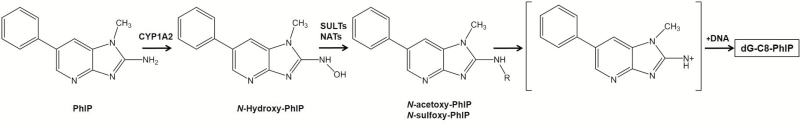
P450-mediated bioactivation pathway of PhIP.

2-Amino-1-methyl-6-phenylimidazo[4,5-*b*]pyridine (PhIP) is one of the most abundantly formed carcinogenic heterocyclic aromatic amines in cooked meat, and it is also present in tobacco smoke (63). Evidence primarily from *in vitro* studies has demonstrated that P450 enzymes are the most important enzymes involved in the initial oxidation of PhIP to form the intermediate *N*-OH-PhIP with CYP1A2 as the predominant P450 enzyme in the activation of PhIP followed by CYP1A1 and 1B1 (64,65) (Figure 3). Initial studies of PhIP activation in *Cyp1a2(−/−*) mice showed that 3 h after exposure to 150 mg/kg bw PhIP administered orally, DNA adduct formation was significantly lower in *Cyp1a2(−/−*) mice compared with wild-type mice in the mammary gland and colon and was not detectable in the liver and kidney of *Cyp1a2(−/−*) mice. These findings indicated the importance of the role of CYP1A2 in the activation of PhIP *in vivo* (66).

In a later study, ~11 or ~22 mg/kg bw PhIP was administered i.p. to *Cyp1a2(−/−*) mice on 8 and 15 days of age, respectively, and 19–21 months later, the hepatic microsomal fractions were isolated (67). The carcinogenic metabolite, *N*-OH-PhIP, was formed by both *Cyp1a2(−/−*) and wild-type hepatic microsomal fractions although *N*-hydroxylation of PhIP was ~8-fold higher in microsomal fractions from wild-type mice than in those from *Cyp1a2(−/−*) mice; this attributes the metabolic activation of PhIP *in vitro* to CYP1A2. However, *Cyp1a2(−/−*) mice administered PhIP had a higher incidence of tumours than wild-type mice (67). The long time interval between carcinogen administration and measurement of microsomal activity should be noted, as this complicates the interpretation of these data.

P450-dependent activation of PhIP was also investigated in the RCN mouse model (40). Hepatic microsomal fractions were isolated from mice exposed to 50 mg/kg bw PhIP orally daily for 5 days, and microsomal incubations with PhIP *in vitro* resulted in 8-fold greater DNA adduct formation with hepatic microsomal fractions from RCN mice without 3MC pretreatment (i.e. mice with active POR) compared with RCN mice with 3MC pretreatment (i.e. mice with inactive POR), implicating a P450-dependent activation mechanism. However, these *in-vitro* results do not correlate with the *in-vivo* findings. DNA adduct formation in extra-hepatic tissues was lower in POR-inactive mice than in POR-active mice, and hepatic DNA adduct formation was not different between both mouse lines. These findings suggest that although Cyp1a2 plays a role in the bioactivation of PhIP *in vitro*, another P450-independent mechanism may also contribute to its activation *in vivo* (40). The *in vivo* findings also somewhat contradict the earlier study carried out with *Cyp1a2(−/−*) mice that found fewer PhIP–DNA adducts in the liver or kidney of *Cyp1a2(−/−*) mice, whereas the levels of adducts in the colon and mammary gland showed little difference from levels in wild-type mice (66). This does offer evidence to the importance of P450-dependent activation mechanisms, however the exposure time is much shorter than in the later studies (40,67).

## 2-Amino-9*H*-pyrido[2,3-*b*]indole

**Figure 4.**
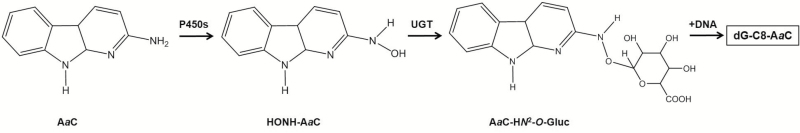
P450-mediated bioactivation pathway of A*a*C.

2-Amino-9*H*-pyrido[2,3-*b*]indole (A*a*C) is another carcinogenic heterocyclic amine present in cooked meat and tobacco smoke. The first step of A*a*C metabolism has been demonstrated in rodent and human liver microsomes to be the *N*-oxidation by P450s to form 2-hydroxyamino-9H-pyrido[2,3-*b*]indole (HONH-A*a*C) that then undergoes conjugation by sulfotransferases or *N*-acetyltransferases (68–70) (Figure 4). To investigate the roles of hepatic and intestinal P450s in the metabolic activation of A*a*C, two mouse models were employed (71). The first mouse model, the liver-specific P450 reductase (*Cpr*)-null mouse model (72), has POR deleted in the liver, whereas the second mouse model, the intestinal epithelium-specific *Cpr*-null mouse, has POR deleted specifically in the intestinal epithelium (73). These two mouse models were compared with wild-type mice to elucidate the roles of hepatic and extrahepatic P450s in A*a*C metabolism. Intestinal epithelium-specific *Cpr*-null mice exposed to 13.6 mg/kg bw A*a*C via gavage did not show any significant differences to wild-type mice in the pharmacokinetic parameters for A*a*C or its metabolites, demonstrating a lack of contribution by intestinal P450s to first-pass clearance of A*a*C. The formation of DNA adducts in intestinal epithelium-specific *Cpr*-null mice was only significantly different from wild-type mice in the bladder where formation was 1.5-fold higher. On the other hand, hepatic microsomal fractions from liver-specific P450 reductase (*Cpr*)-null mice were unable to oxidize A*a*C *in vitro,* whereas DNA adduct formation *in vivo* was found to be not lower in liver than in wild-type mice and significantly higher in lung (4-fold), bladder (1.2-fold) and colon (4-fold) (71). These findings suggest that P450 enzymes do not contribute significantly to the activation of orally administered A*a*C, but they may play a greater role in its detoxication (71).

## 4-(Methylnitrosamino)-1-(3-pyridyl)-1-butanone

**Figure 5.**
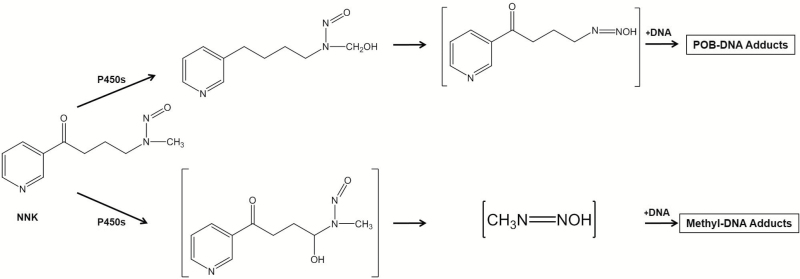
P450-mediated bioactivation pathway of NNK.

4-(Methylnitrosamino)-1-(3-pyridyl)-1-butanone (NNK) is one of the most potent carcinogens present in tobacco smoke. Carcinogenesis occurs with P450-mediated α-hydroxylation producing reactive metabolites that induce the formation of pyridyloxobutyl- (POB-) and methyl-DNA adducts such as O^6^-methylguanine (O^6^-mG) (74–76) (Figure 5). This has been demonstrated *in vivo* with the use of P450 inhibitors (77,78) and *in vitro* with lung microsomes (74). Although activation of NNK is carried out in the lungs, hepatic microsomes have been shown to be at least as active as lung microsomes in activating NNK *in vitro* (79). To further elucidate the role of hepatic and pulmonary P450s, two mouse models were used, a lung-*Cpr*-null mouse (i.e. POR null) generated by cross-breeding *CCSP-rtTA/tetO-Cre* mice and *Cpr*^*lox/lox*^ mice that allows doxycycline-inducible lung-specific *Cpr* deletion (80), and a liver-*Cpr*-null mouse generated by breeding pairs of hemizygous Alb-Cre transgenic mice, with Cre driven by the albumin promoter that lack POR expression in the liver (72). A single dose of 10 and 20 µmol/mouse NNK was administered i.p. to liver-*Cpr*-null and lung-*Cpr*-null mice, respectively, and mice were sacrificed 4 months later. The lung-*Cpr*-null mice had fewer lung tumours than wild-type mice, implicating the role of pulmonary P450 enzymes in the activation of NNK. The liver-*Cpr*-null mice, however, had ‘higher’ levels of lung tumour multiplicity than wild-type mice (80).

These findings were confirmed in a later study using HRN-*gpt* delta mice (44). After exposure to a single i.p. dose of 100 mg/kg bw NNK, NNK-induced mutation frequency was three times higher in the lung of HRN-*gpt* delta mice than in control *gpt* delta mice. Furthermore, pharmacokinetic studies showed significantly higher plasma levels of NNK and significantly lower rates of clearance in HRN-*gpt* delta mice compared with controls, suggesting that although pulmonary P450 enzymes play a role in NNK activation, hepatic P450 enzymes may in fact play a larger role in detoxication (44). However, *in-vitro* studies using recombinant CYP2A5 demonstrated the ability of this CYP isoenzyme to efficiently activate NNK (75,81), and NNK-induced lung tumorigenesis in mice was found to be reduced when CYP2A enzymes were inhibited (82). When *Cyp2a5*-null mice were given a single i.p. dose of NNK at 20 or 100 mg/kg bw, there was no change in the systemic clearance of NNK or its major circulating metabolite 4-(methylnitrosamino)-1-(3-pyridyl)-1-butanol (NNAL). Levels of pulmonary O^6^-mG adducts were significantly lower, ~40% at 20 mg/kg and ~20% at 100 mg/kg, in *Cyp2a5*-null mice compared with wild-type mice. Levels of hepatic O^6^-mG adducts, however, showed no significant differences between *Cyp2a5*-null mice and wild-type mice at either dose despite previous studies showing that hepatic P450s were protective against NNK-induced lung tumorigenesis (80,83).

The knockout was extended to *Cyp2abfgs*-null mice, in which all Cyp2a, 2b, 2g, 2f and 2s genes are deleted, with i.p. doses of 50 or 200 mg/kg bw NNK and sacrifice 16 weeks post exposure (84). Levels of pulmonary O^6^-mG adducts were substantially reduced compared with both wild-type and *Cyp2a5*-null mice 1 and 4 h post exposure, with hepatic O^6^-mG adduct levels only showing a significant reduction 4 h post exposure to 200 mg/kg bw NNK. The *Cyp2abfgs*-null mice also demonstrated resistance to NNK-induced lung tumorigenesis at both the low and high NNK doses, unlike the wild-type or *Cyp2a5*-null mice. In contrast to the POR-knockout mice, these results suggest that there is in fact a contribution from mouse CYP2A/B/F/G/S enzymes toward the bioactivation of NNK *in vivo* (84).

## Conclusions

The role of P450 enzymes in the metabolism of environmental carcinogens is complex. While numerous *in-vitro* studies have demonstrated the role of P450s in the activation of carcinogens, the use of *Cyp*-knockout mice or *Por*-knockout mouse models lacking P450 enzyme activity has yielded paradoxical results demonstrating that P450 enzymes *in vivo* are in fact more important for detoxication. Although P450s are capable of activating carcinogens to their reactive intermediates, in an *in vivo* situation where myriad more biological factors are at play, this role appears to shift. These results are also not limited to a particular carcinogen or carcinogenic family, or to a particular knockout mouse model. Although the numerous mouse models utilized have been derived by a variety of methods for disrupting P450 expression or activity (9,10), there is the possibility that by disrupting P450 expression or activity, the metabolic balance in tissues is disturbed and alternative contributing factors to activation or detoxification that may have been minor come to the fore. Many of the studies discussed have short exposure times (<1 month) and have focused on short-term markers, e.g. protein or DNA adduct and metabolite formation, which may not reflect the longer term carcinogenic consequences. Nevertheless, two of the studies described here have investigated tumour formation with different carcinogens and have yielded the same overall paradoxical outcome as the short-term studies (67,80). Across the different target organs for the carcinogens, there is no overlap in any single organ for all the carcinogens in experimental mice: (i) exposure to BaP results in malignant tumours in the lung, forestomach, liver, lymphoid tissue and skin (85); (ii) 4-ABP causes bladder carcinoma in male mice and hepatocellular carcinoma in female mice (86); (iii) PhIP causes tumours in the lungs and lymphatic systems (87); (iv) A*a*C leads to tumours in the livers (87,88) and (v) NNK and leads to tumours in the lung and forestomach of experimental mice (89,90).

While *Por*-knockout mouse models lacking P450 enzyme activity are powerful tools for investigations of xenobiotic metabolism, the paradoxical results they yield require further investigations to better understand mechanisms of activation. One possibility that arises from these studies is the potential for P450-independent activation pathways that are as yet unidentified.

## Funding

Lindsay Reed is supported by a King’s College London Health Faculty PhD Studentship funded by the Medical Research Council (grant 1524896). Work at King’s College London is supported by Cancer Research UK (Grant C313/A14329), Wellcome Trust (Grants 101126/Z/13/Z and 101126/B/13/Z), Natural Environment Research Council (Grant NE/L006782/1) and in part by the National Institute for Health Research Health Protection Research Unit (NIHR HPRU) in Health Impact of Environmental Hazards at King’s College London in partnership with Public Health England (PHE). The views expressed are those of the authors and not necessarily those of the NHS, the NIHR, the Department of Health or PHE.

## Conflict of Interest Statement

None declared.

## Abbreviations

AaC: 2-amino-9H-pyrido[2,3-b]indole
AHR: aryl hydrocarbon receptor
BaP: benzo[a]pyrene
BPDE: BaP-7,8-dihydrodiol-9,10-epoxide
bw: body weight
Cyb5: cytochrome b5
Cyb5R: cytochrome b5 reductase
CYP: cytochrome P450
HBRN: hepatic cytochrome b5/P450 reductase null
HRN: hepatic cytochrome P450 reductase null
NNAL: 4-(methylnitrosamino)-1-(3-pyridyl)-1-butanol
NNK: 4-(methylnitrosamino)-1-(3-pyridyl)-1-butanone
O^6^-mG: O^6^-methylguanine
PhIP: 2-amino-1-methyl-6-phenylimidazo[4,5-b]pyridine
POR: cytochrome P450 reductase
RCN: reductase conditional null
TCDD: tetrachlorodibenzo-*p*-dioxin
